# Genetically-increased taste cell population with Gα-gustducin-coupled sweet receptors is associated with increase of gurmarin-sensitive taste nerve fibers in mice

**DOI:** 10.1186/1471-2202-10-152

**Published:** 2009-12-22

**Authors:** Keiko Yasumatsu, Tadahiro Ohkuri, Keisuke Sanematsu, Noriatsu Shigemura, Hideo Katsukawa, Noritaka Sako, Yuzo Ninomiya

**Affiliations:** 1 Section of Oral Neuroscience, Graduate School of Dental Science, Kyushu University, Fukuoka 812-8582, Japan; 2 Department of Oral Physiology, Asahi University School of Dentistry, Gifu 501-0296, Japan

## Abstract

**Background:**

The peptide gurmarin is a selective sweet response inhibitor for rodents. In mice, gurmarin sensitivity differs among strains with gurmarin-sensitive C57BL and gurmarin-poorly-sensitive BALB strains. In C57BL mice, sweet-responsive fibers of the chorda tympani (CT) nerve can be divided into two distinct populations, gurmarin-sensitive (GS) and gurmarin-insensitive (GI) types, suggesting the existence of two distinct reception pathways for sweet taste responses. By using the *dpa *congenic strain (*dpa CG*) whose genetic background is identical to BALB except that the gene(s) controlling gurmarin sensitivity are derived from C57BL, we previously found that genetically-elevated gurmarin sensitivity in *dpa *CG mice, confirmed by using behavioral response and whole CT nerve response analyses, was linked to a greater taste cell population co-expressing sweet taste receptors and a Gα protein, Gα-gustducin. However, the formation of neural pathways from the increased taste cell population to nerve fibers has not yet been examined.

**Results:**

Here, we investigated whether the increased taste cell population with Gα-gustducin-coupled sweet receptors would be associated with selective increment of GS fiber population or nonselective shift of gurmarin sensitivities of overall sweet-responsive fibers by examining the classification of GS and GI fiber types in *dpa *CG and BALB mice. The results indicated that *dpa CG*, like C57BL, possess two distinct populations of GS and GI types of sweet-responsive fibers with almost identical sizes (*dpa CG*: 13 GS and 16 GI fibers; C57BL: 16 GS and 14 GI fibers). In contrast, BALB has only 3 GS fibers but 18 GI fibers. These data indicate a marked increase of the GS population in *dpa *CG.

**Conclusion:**

These results suggest that the increased cell population expressing T1r2/T1r3/Gα-gustducin in *dpa CG *mice may be associated with an increase of their matched GS type fibers, and may form the distinct GS sweet reception pathway in mice. Gα-gustducin may be involved in the GS sweet reception pathway and may be a key molecule for links between sweet taste receptors and cell type-specific-innervation by their matched fiber class.

## Background

Gurmarin (Gur) is a peptide isolated from a plant, gymnemma sylvestre. This peptide was shown to selectively inhibit the taste responses to sweet substances without affecting the responses to other basic taste stimuli, such as NaCl, HCl and quinine in rodents [1-4]. In mice, the Gur sensitivity differs among tongue regions and strains [2-4]. That is, the Gur inhibition of whole nerve integrated responses to sweet compounds is clearly evident only in the chorda tympani (CT) nerve innervating the anterior tongue, but not in the glossopharyngeal nerve innervating the posterior tongue. The responses of the CT nerve to sucrose (0.01 - 1.0 M) significantly decrease to about ~50% of control after Gur treatment in C57BL but only slightly if at all in BALB/c (BALB) mice [2,5]. In C57BL mice, sweet-responsive CT fibers can be classified into two distinct populations, Gur-sensitive (GS) and Gur-insensitive (GI) types, suggesting that there may be at least two distinct reception pathways for mouse sweet responses [6]. However, potential factors involved in the formation of the two distinct pathways from taste cells to axons remain largely unknown.

Previous molecular studies revealed that sweet taste receptors are composed of taste receptor type 1, member 2 (T1r2) and 3 (T1r3) heterodimers [7-15]. These dimers are coupled with guanine nucleotide-binding proteins (G proteins), such as Gα-gustducin, Gαi-2 and Gαs, and lead to downstream signaling for sweet reception[16,17] Recently, it has been proposed that differences in Gur sensitivity between mouse strains and tongue regions may be related to differences in the co-expression patterns of T1r2/T1r3 and Gα-gustducin in taste cells [18]. This proposal is supported by the following: in GS fungiform papillae in the anterior tongue, T1r2-positive cells co-expressed both T1r3 and Gα-gustducin, whereas T1r2 and T1r3 double-positive cells rarely expressed Gα-gustducin in GI circumvallate papillae in the posterior tongue[18,19]. The ratio of cell expressing all three genes (T1r2/T1r3/Gα-gustducin) is greater in the order of fungiform papillae of GS C57BL mice > fungiform papillae of Gur-weakly-sensitive BALB mice > GI circumvallate papillae in C57BL and BALB mice [18]. This indicates that lower sensitivity to Gur in BALB fungiform papillae may be associated with a lower co-expression ratio of the three genes. The lack of Gur sensitivity in circumvallate papillae of C57BL mice may be associated with their lack of co-expression between T1r2/T1r3 receptors and Gα-gustducin. Recent studies demonstrated that the GI circumvallate taste bud cells expressing T1r2/T1r3 receptors, instead, co-expressed Gα14 [20,21]. Moreover, in our previous study using the *dpa *congenic strain (*dpa CG*) [5,22-24] whose genetic background is identical to BALB except that the gene(s) controlling Gur sensitivity are derived from C57BL, we found that the co-expression level between T1r2/T1r3 and Gα-gustducin in the fungiform taste bud cells of *dpa CG *mice is almost identical to that of GS C57BL mice [18]. Thus, the genetically-elevated Gur sensitivity in *dpa CG *may be associated with a larger taste cell population co-expressing sweet taste receptors and Gα-gustducin. Our more recent study using Gα-gustducin-null mutant mice demonstrated that their residual CT responses to sucrose and glucose are not suppressed by Gur [25]. These results suggest the possibility that Gα-gustducin may be involved in the GS reception system and may act as a key molecule linking between sweet taste receptors and possible cell type-specific-innervation by matched fiber class.

As the first step to test this possibility, we investigated whether the genetically-increased taste cell population with Gα-gustducin-coupled sweet receptors in *dpa CG *would be associated with a selective increment of the GS fiber population or a nonselective shift of gurmarin sensitivity of the entire population of sweet-responsive fibers. In the former case, we would expect to identify two distinct GS and GI populations as is the case in C57BL mice, whereas in the latter there would be one single population with broad Gur sensitivities peaking at a level intermediate between those for GI and GS types. To accomplish this, we classified the single CT nerve fibers according to their Gur sensitivity in *dpa CG *and their inbred partner strain, BALB, and then compared them with previously obtained data from C57BL mice. We found that *dpa CG*, like C57BL, possessed two distinct populations of GS and GI types of sweet-responsive fibers with almost identical size, indicating a marked increase of the GS population as compared with BALB mice. These results suggest that the increased cell population expressing T1r2/T1r3/Gα-gustducin in *dpa CG *mice may be associated with an increase of their matched GS type fibers. Gα-gustducin may be a key molecule involved in the GS sweet reception pathway and may play a role in links between sweet taste receptors and cell type-specific-innervation by their matched fiber class.

## Methods

### Animals

All experimental procedures were approved by the committee for Laboratory Animal Care and Use at Kyushu University (Fukuoka, Japan). Subjects were adult male and female BALB/cNCrj mice [BALB, 8-16 weeks of age, ranging in weight from 23 to 34 g, obtained from Charles River (Tokyo, Japan)], and *dpa *congenic mice [*dpa CG*, 8-16 weeks of age, ranging in weight from 23 to 35 g]. The *dpa CG *line was established using standard techniques [22-24]. The segment of the chromosome of the sweet taster C57BL/6NCrj (C57BL:donor), carrying the gene responsible for sweet responses to D-phenylalanine (D-Phe), was transferred onto non-sweet taster BALB (partner) background by continuous backcrossing. The phenotype for D-Phe sweet-taster in each generation was selected by using a conditioned taste aversion paradigm for behavioral measurement of the taste similarities between 0.03 and 0.1 M D-Phe and 0.1 M sucrose. Behavioral responses (conditioned avoidance responses) of this *dpa CG *strain to D-Phe and its generalization to other sweeteners were not significantly different from those of the donor C57BL strain. Our recent study revealed that responses to sweet substances and Gur sensitivity in *dpa CG *mice of N16 generation (15 times back-crossing) were almost identical to those of donor C57BL mice in both behavioral experiments using a short term lick test and electrophysiological experiment recording taste responses from the whole CT nerve (not from single CT fibers) [5,26]. The *dpa CG *is a Gur-sensitive strain with almost the same genetic background as the Gur-weakly- sensitive BALB strain [5]. In the present study, we used the *dpa *CG of generation N16 (99.99694% of their genetic background is calculated to be identical to that of the BALB strain).

### Recordings of responses from single CT nerve fibers

The procedures for dissection and recording of responses from the CT nerve fibers were the same as those used previously [6,27,28]. Under pentobarbital anesthesia (40-50 mg/kg ip), the trachea of each mouse was cannulated, and the mouse was then fixed in the supine position with a head holder to allow dissection of the CT nerve. The right CT nerve was dissected free from surrounding tissues after removal of the pterygoid muscle and cut at the point of its entry into the bulla. For single-fiber recording, a single or a few fibers of the nerve were teased apart with a pair of needles and lifted onto an Ag-AgCl electrode. An indifferent electrode was placed in nearby tissue. Impulse discharges resulting from chemical stimulations of the tongue were fed into an amplifier (K-1; Iyodenshikogaku, Nagoya, Japan), and monitored on an oscilloscope and audiomonitor, recorded on a computer for later analysis using a PowerLab system (PowerLab/sp4; AD Instruments, Australia).

### Chemical stimulations of the tongue

The anterior half of the tongue was enclosed in a flow chamber made of silicone rubber [29]. Solutions were delivered into the chamber by gravity flow and flowed over the tongue for a controlled period. Solutions used as chemical stimuli were: 0.1 M NaCl, 0.01 M HCl, 0.02 M quinine HCl, 0.5 M sucrose, 20 mM saccharin Na, 0.1 M D-Phe (Wako Pure Chemicals Industries, Osaka, Japan). These chemicals were dissolved in distilled water and used at ~24°C. The order of chemical stimulation during the first survey to find fibers responding to sweet compounds was sucrose, NaCl, HCl and quinine. If the fiber clearly responded to sucrose, we continued and further applied other sweet substances. Then sucrose was applied once more to check the reproducibility of the response before the lingual treatment with Gur. During chemical stimulation of the tongue, the test solution flowed for ~30 s at the same flow rate as the distilled water used for rinsing the tongue (~0.1 ml/s). The tongue was rinsed with distilled water for an interval of ~1 min between successive stimulations. To examine Gur inhibition of the CT responses, the tongue was treated with 30 μg/ml (~7.13 μM) Gur dissolved in 5 mM phosphate buffer (pH 6.8; made with Na_2_HPO_4_12H_2_O and NaH_2_PO_4_2H_2_O) for 5 min. Gur is reported to be very stable against cleavage by proteases, even under conditions such as high temperature, low pH, and presence of urea [30]. The inhibitory effects of Gur on rat CT responses to sucrose were hardly changed when the Gur solution was heated to 90°C, and stored for ≥ 2 mo [1]. To obtain similar potencies of Gur throughout the experiments, we used aliquots of the same stock solution of Gur that were stored at -20°C and warmed to room temperature immediately before application to the tongue. We chose a concentration of Gur (30 μg/ml: 7.13 μM) that was about 50% higher than the concentration (20 μg/ml: 4.8 μM) that exhibited the maximum suppressive effect on CT responses in C57BL mice [2,6]. Gur was applied to the tongue only once, and therefore data from only one preparation were obtained from each animal. In some fibers in which sucrose responses were suppressed by Gur, to facilitate the recovery of the suppressed sweetener responses, the animal's tongue was rinsed for 10 min with 15 mM β-cyclodextrin (β-CD), which could remove the effect of Gur [31]. In these fibers, after the Gur and β-CD experiments, 2% Pronase E (Pronase: dissolved in 50 mM phosphate buffer at pH 6.8), a specific inhibitor for sweetener responses in rats [32] and mice [3,6], was further applied for 10 min to the tongue to check its inhibitory effects on sweetener responses.

### Data analysis

For recordings from multiple taste fibers, the responses of single fibers were segregated with the help of spike wave form analysis (PowerLab/sp4; AD Instruments, Australia). We used waveform shape parameters (width, height, peak amplitude, antipeak amplitude, interspike interval) to segregate each single unit [27,28]. Frequency-time histograms of impulse discharges before, during, and after chemical stimulation of the tongue were calculated by means of spike-analysis programs (SAS-1; Iyodensikogaku, Nagoya, Japan; Spike histogram; AD Instruments, Australia). For data analysis, we used the net average frequency for the first 5 sec after the stimulus onset, which was obtained by subtracting the spontaneous frequency for the 5 sec period before stimulation. The final criteria for the occurrence of a response were the following: the number of spikes was larger than the mean plus 2 SDs of the spontaneous discharge in two repeated trials, and at least +3 spikes were evoked by taste stimulation. Thus for fibers without any spontaneous discharge or with very low rates of spontaneous discharge, 3 spikes was considered a response. We calculated the percentage of Gur inhibition of the response to 0.5 M sucrose for each fiber and used the 60% control level to classify the fibers as either GS-type (< 60%) or GI-type (≥ 60%). Our previous study has shown that the 60% level was the most appropriate boundary for classifying the two groups of fibers with different Gur sensitivity in mice [6]. Data for C57BL mice were obtained from our previous study [6] (number of fibers = 30).

## Results

**Figure 1:**
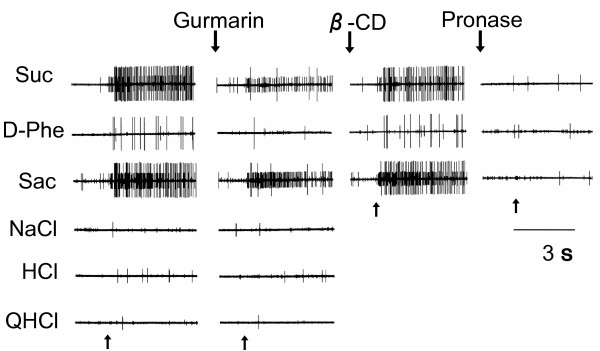
**Sample recordings from two different sweet-responsive fibers of a *dpa CG *mouse with one large GS unit and one small GI unit**. The recordings include responses of the two units to 0.5 M sucrose (Suc), 0.1 M D-Phe and 20 mM saccharin (Sac), 0.1 M NaCl, 0.01 M HCl and 0.02 M quinine HCl (QHCl) before and after treatment with 7.13 μM Gur for 5 min, and responses to Suc, D-Phe and Sac after rinsing the tongue with 15 mM β-CD for 10 min and after further treatment with 2% Pronase for 10 min. Stimulation started at the point indicated by the arrow.

In the present study, we tested the responses to 0.5 M sucrose of in total 90 single CT fibers for each strain and obtained 29 and 21 sucrose-responsive fibers from *dpa *CG and BALB mice, respectively. Fig 1 shows recordings of taste responses obtained from two different sweet-responsive fibers from a *dpa CG *mouse with one large GS unit and one small GI unit before and after treatment with Gur, β-CD and Pronase. Usually we dissected the taste fibers into fine strands so that we can record the responses from one single unit. But occasionally, responses from two differential fibers were obtained. In the recording shown in Fig. 1, the large fiber responded robustly to all three sweet stimuli, 0.5 M sucrose, 0.1 M D-Phe and 20 mM saccharin, whereas the small one responded to sucrose and saccharin but not to D-Phe. Both fibers did not respond to the other basic taste stimuli, NaCl, HCl and quinine. The large fiber exhibited clear suppression of impulse discharges to sweet compounds after application of Gur, and recovery after subsequently rinsing the tongue with β-CD, indicating a GS fiber. In contrast, the small fiber showed no such suppression of sweet responses by Gur, indicating a GI fiber. The responses to sweet compounds in both fibers were totally abolished after application of Pronase, a sweet response inhibitor, indicating that the responses to sweet compounds in both fibers occur through activation of Pronase-sensitive sweet-responsive elements on the apical membrane of taste receptor cells.

**Figure 2:**
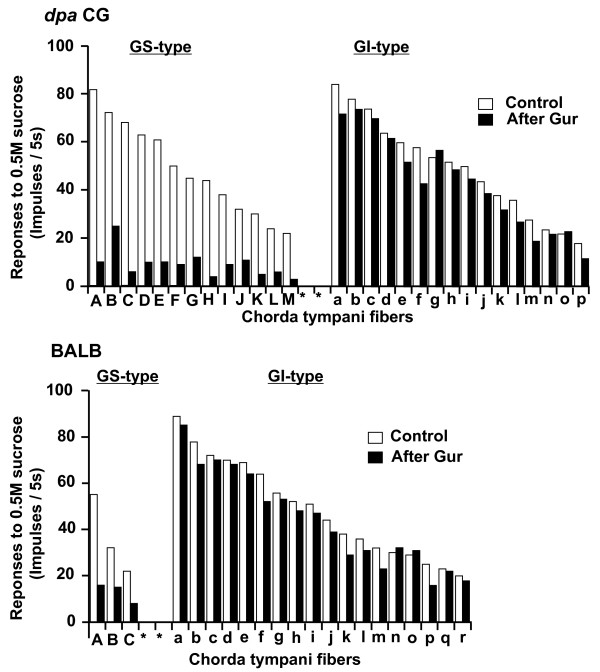
**Distributions of sucrose responses of the GS-type and GI-type of chorda tympani fibers in *dpa *CG and BALB mouse strains**. Fibers were classified into GS-type (*dpa *CG: A-M; BALB: A-C) and GI-type(*dpa *CG: a-p; BALB: a-r) based on inhibition of responses (impulses/5 sec) to 0.5 M sucrose by 7.13 μM Gur. Open columns indicate responses before Gur, while filled columns exhibit responses after Gur.

**Figure 3:**
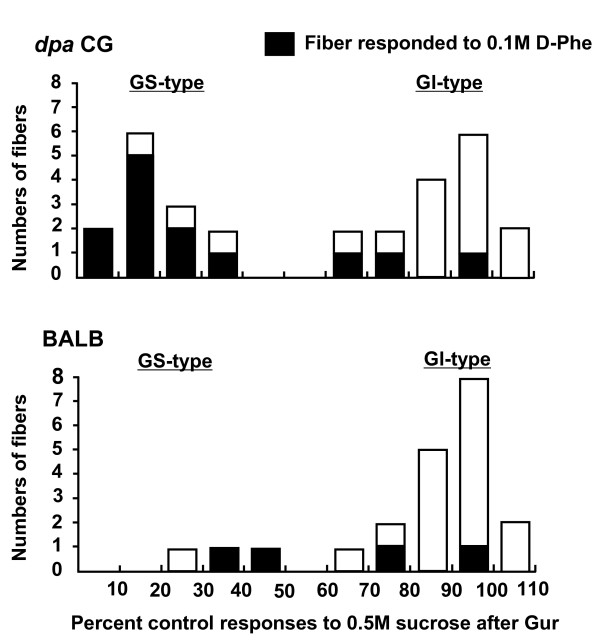
**Distributions of GS- and GI-type chorda tympani fibers of *dpa *CG and BALB mouse strains across their percent control responses to 0.5 M sucrose after Gur**. Control responses before treatment with 7.13 μM Gur present as 100%. In each column, the filled part indicates the number of fibers responding to 0.1 M D-Phe.

In *dpa CG *mice, in total 29 fibers responding to sucrose were sampled. Out of those 29, 13 fibers exhibited suppression of responses to 0.5 M sucrose by Gur to < 50% of control (Fig. 2-*dpa CG*, GS), indicating the existence of a GS fiber population made of about 45% of all sweet-sensitive fibers. The percent control responses of this population to 0.5 M sucrose after Gur ranged from 8.8 to 34.7% with a mean of 19.7 ± 8.7% (SD; Fig. 2-*dpa CG*, GS; Fig.3-*dpa CG*, GS). The mean number of impulses/5 s of the 13 GS fibers in response to 0.5 M sucrose after Gur (9.2 ± 5.5) was significantly smaller than that before Gur (48.6 +19.3, t-test, P < 0.001; Fig. 2-*dpa CG*, GS, A-M). In the remaining 16 fibers, the sucrose responses were only slightly if at all inhibited by Gur (Fig. 2-*dpa CG*, GI, a-p). The percent control responses of this population to 0.5 M sucrose after Gur ranged from 66.7 to 105.6% with a mean of 87.6 ± 11.7% (Fig.3-*dpa CG*, GI). The mean number of impulses/5 s of the 16 GI fibers to 0.5 M sucrose after Gur (43.6 ± 19.9) was not significantly different from that before Gur (49.0 ± 20.4, paired t-test, P > 0.05; Fig. 2-*dpa CG*, GI, a-p).

In BALB mice, in total 21 fibers responding to sucrose were sampled. Out of those 21, only 3 fibers exhibited reduction of impulse discharges to 0.5 M sucrose after Gur to < 50% of control (Fig.2-BALB, GS). The percent control responses of the 3 GS fibers to 0.5 M sucrose after Gur ranged from 29.1 to 46.9% with a mean of 37.4 ± 8.9% (Fig.3-BALB, GS). The mean number of impulses/5 s of the 3 GS fibers to 0.5 M sucrose after Gur (13.0 ± 3.5) was significantly smaller than that before Gur (36.3 ± 13.8, t-test, P < 0.05; Fig. 2-BALB, GS, A-C). In the remaining 18 fibers, the sucrose responses were only slightly if at all inhibited by Gur (Fig. 2-BALB, GI, a-r). The percent control responses of this population to 0.5 M sucrose after Gur ranged from 64.0 to 106.9% with a mean of 89.9 ± 11.1% (Fig. 3-BALB, GI). The mean number of impulses/5 s of the 18 GI fibers to 0.5 M sucrose after Gur (44.2 ± 20.0) was not significantly different from that before Gur (48.8 ± 21.1, t-test, P > 0.05; Fig. 2-BALB, GI, a-r). Although the 3 fibers were classified into GS type in BALB mice, the mean sucrose response after Gur in the total 21 fibers (47.0 ± 20.6 impulses/5 s) was not significantly different from that before Gur (39.8 ± 22.1). In this sense, the present data from single fiber experiments is comparable to the data from the previous study using a whole nerve response analysis [2].

Fiber distributions across percent control response after Gur in two strains are shown in Fig.3. The distribution patterns clearly indicate that *dpa CG *mice possess two distinct populations of sweet-responsive fibers, GS and GI types. The numbers of GS (13) and GI (16) fibers in *dpa CG *mice did not significantly differ from those of C57BL mice (16 GS and 14 GI fibers), reported previously [6] (Chi-squre = 0.5, P > 0.05). The *dpa CG *strain possesses markedly increased GS population as compared to BALB mice, their parent inbred partner strain (3 GS and 18 GI fibers; Chi-square = 5.2, P < 0.05). The total number of fibers tested for *dpa CG *and BALB mice was 90. The ratio of sweet-responsive fibers (29 for *dpa CG *and 21 for BALB) vs non-sweet-responsive fibers (61 for *dpa CG *and 69 for BALB) did not significantly differ between *dpa CG *and BALB mice (Chi-square = 2.5, P > 0.05), indicating no change in entire population size for sweet-responsive fibers in *dpa CG *mice. Thus, the relative population size of GS vs GI type increased in *dpa CG *mice. It is also noted that most of the GS fibers of these strains responded to D-Phe, suggesting a strong relationship between sensitivities to Gur and D-Phe, as shown in C57BL mice previously [6].

**Figure 4:**
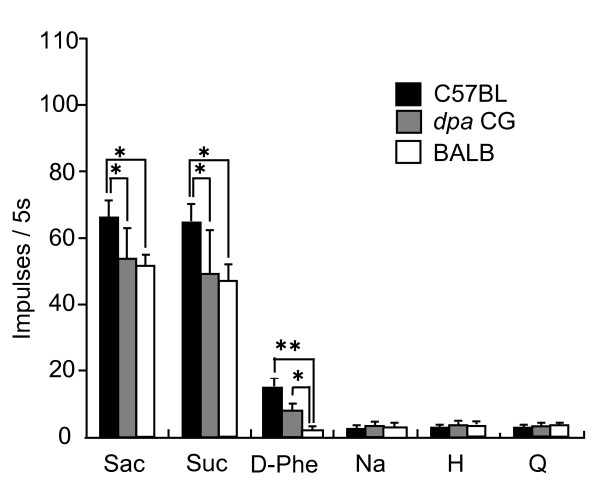
**Mean numbers of impulses/5 sec of all sweet-sensitive fibers including GS and GI types in response to 6 taste stimuli in C57BL, *dpa CG *and BALB mice**. Two-way repeated ANOVA tests indicated that the mean number of impulses to Sac, Suc and D-Phe significantly differed among the 3 strains [F (2, 77) = 3.70, P < 0.05 for Sac; F (2, 77) = 4.52, P < 0.05 for Suc; F (2, 75) = 5.52, P < 0.01 for D-Phe]. *: Post-hoc Turkey-Kramer tests: P < 0.05. Data for C57BL presented were obtained by recalculation of those from Ninomiya et al. (1999)[6]

Fig. 4 presents the mean numbers of impulses/5 sec of all sweet-sensitive fibers including GS and GI types to 6 taste stimuli in C57BL, *dpa CG *and BALB mice. Two-way repeated ANOVA tests indicated that the mean impulses in response to Sac, Suc and D-Phe significantly differed among the 3 strains [F (2,77) = 3.70, P < 0.05 for Sac; F (2, 77) = 4.52, P < 0.05 for Suc; F (2, 75) = 5.52, P < 0.01 for D-Phe], whereas no such differences were observed in the responses to other non-sweet stimuli (F = 0.09 - 0.66, P > 0.05). Post-hoc Tukey-Kramer tests suggest that the number of impulses in response to Suc and Sac in *dpa CG *did not significantly differ from those in BALB, but was smaller than that in C57BL mice (P < 0.05). The number of impulses to D-Phe in *dpa CG *mice was significantly larger than that in BALB mice (P < 0.05). This suggests that the sensitivities to sweet substances except D-Phe in each fiber in *dpa CG *mice might not largely differ from those in BALB mice. Collectively, the present study revealed that *dpa CG *mice have an elevated size of population of GS sweet fibers which is almost identical to that of C57BL mice.

## Discussion

In the present study, we used the *dpa *congenic strain (*dpa CG*) [22-24] whose genetic background is identical to BALB except gene(s) controlling Gur sensitivity derived from C57BL. Gur inhibition of sweet responses in *dpa *CG at this N16 generation has been confirmed by behavioral response analysis using a short term lick test and a whole CT nerve response analysis [5,25]. Increased responses to D-Phe of sweet-responsive fibers in *dpa CG *as compared to BALB, which may be due to an increase of D-Phe-sensitive GS type fibers [6], were further confirmed in the present study (Fig.4). Our previous study examined the genotypes of *Tas1r3 *in *dpa CG *mice by using restriction enzymes, and the results suggested that the *Tas1r3 *gene of *dpa CG *may be derived from BALB (*Tas1r3 *non-taster genotype), but not from C57BL (*Tas1r3 *taster genotype) mice [5]. Therefore, the responses to Suc and Sac in *dpa *CG in this study were not significantly different from those in BALB mice, but were lower than those of C57BL mice (Fig.4).

In the present study, we investigated if the genetically-increased cell population co-expressing sweet receptors and Gα-gustducin in *dpa CG *would be associated with an increase in GS type fibers by examining the classification of single CT fiber types of *dpa CG *mice according to their Gur sensitivities, and then comparing them with those of C57BL and BALB mice. We used Gur at a concentration [30 μg/ml (7.13 μM)] that was 50% higher than the concentration (20 μg/ml) sufficient to produce maximum suppression of the sucrose response in C57BL mice [2]. Under these conditions, where any GS responses would be maximally inhibited, we found that in *dpa CG *mice 29 sweet-responsive fibers sampled were classified into 13 GS and 16 GI fibers (Fig.3- *dpa CG*), whereas in BALB mice, 21 sweet-responsive fibers were classified into 3 GS and 18 GI fibers (Fig.3-BALB). Our previous study revealed that in C57BL mice 30 sweet-responsive fibers were classified into 16 GS and 14 GI type fibers [6]. Sweet-responsive GS and GI fibers were sampled from in total 90 CT fibers responding to at least one taste stimulus in each strain. Thus, *dpa *CG mice, like C57BL, possess two distinct fiber populations (GS and GI) with almost identical size, indicating a marked increase of the GS population as compared to BALB mice. This suggests that the increased cell population co-expressing T1r2, T1r3 and Gα-gustducin in *dpa CG *mice may be associated with an increase of their matched GS type fibers, and may form the distinct GS reception pathway in mice.

Differential features of the GS and GI sweet-reception systems in mice have also been found in their reformation process during regeneration of the mouse CT nerve after nerve crush [4]. That is, responses to sweet compounds reappeared at 3 weeks after the CT nerve crush. At this period, sweet responses were not suppressed by Gur, indicating reappearance of only the GI response component. Gur-inhibition of sweet responses was clearly observed from 4 weeks onward, indicating that recovery of the GI component preceded recovery of the GS component by about 1 week. Single CT fibers responsive to sucrose could be classified into GS or GI types at 4 weeks. This indicates that GS and GI taste cells may be selectively innervated by their matching GS and GI type taste axons, respectively, and then reform two separate signaling pathways for sweet taste during regeneration. In the previous study, expression of Gα-gustducin and T1r3 mRNAs was observed in taste bud cells already at 2 weeks after nerve crush and the number of cells expressing mRNA of these genes gradually increased after that. Thus, there may be a cell population expressing T1r3 and/or Gα-gustducin already before reformation of the GS neural pathway, although there is no evidence for consistency between functional maturation of the reception systems through these molecules and reformation of the GS neural pathway.

**Figure 5:**
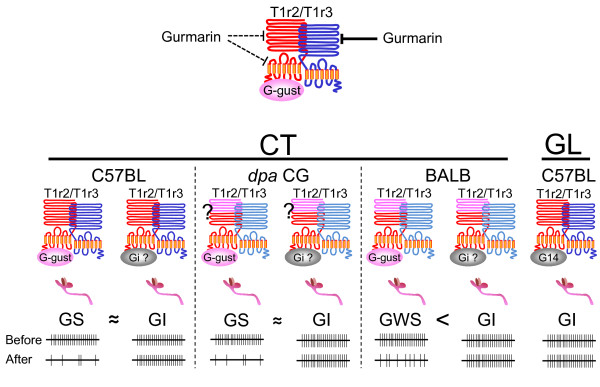
**Speculative models for the Gur binding sites of T1r2/T1r3 sweet receptor and GS and GI receptor and neural systems in 3 strains of mice (C57BL, BALB and *dpa *CG)**. Upper panel: a major binding site of Gur may be the extracellular domain of the T1r3. Gur may also possess minor binding sites at the extracellular and transmembrane domains of T1r2. Lower panels: the GS system of C57BL mice may possess Gur binding sites at both extracellular and transmembrane domains of T1r2 in addition to its major binding site for T1r3, whereas the Gur-weakly-sensitive (GWS) system of BALB may possess a Gur binding site at only the transmembrane domain of T1r2 in addition to the extracellular domain of T1r3. It remains unclear whether *dpa *CG may possess the GS system or the GWS system, although in this figure we tentatively label it as the GS system. In all three strains, the GI system may have Gα proteins other than Gα-gustducin in the anterior tongue innervated by the chorda tympani (CT) and posterior tongue innervated by the glossopharyngeal (GL) nerves. The proportion of GS vs GI components in the anterior tongue may be dependent on co-expression rates of Gα-gustducin and T1r2/T1r3; C57BL and *dpa *CG mice possess almost identical sizes of GS and GI population, whereas BALB mice possess a much larger size of GI population.

The effect of Gur is normally long-lasting (> 1 h) but rapidly disappears after rinsing the tongue with either anti-Gur serum in rats [33] or β-CD in mice as shown in Fig.1. This quick recovery from inhibition by Gur by rinsing the tongue with the Gur-binding agents indicates that Gur can directly interact with sweet taste receptors, such as T1r2/T1r3, on the taste membrane in rodents. One cell-based assay using human-embryonic-kidney 293 cells expressing various combinations of human and mouse T1r2/T1r3 chimeras indicated that Gur may interact with the extracellular domain of mouse T1r3 [34]. This is consistent with the finding shown in our previous study that the order of diminution of CT responses to various sweet compounds in mice genetically lacking T1r3, SC45647 (~95% of control) > sucrose (~80%) > maltose (~50%) > glucose and sorbitol (~30%) is similar to that by Gur in wild type C57BL mice, SC45647 (~80% of control) > sucrose, maltose (~50%) > glucose and sorbitol (~30%) [4,25,35]. Therefore, it may be that Gur inhibition of sweet receptor activity would start with binding of Gur to the extracellular domain of T1r3, which may produce conformational changes of the T1r2/T1r3 receptor, resulting in abolishment or severe loss of its ligand binding affinity in some ligand-specific manner. However, T1r3 may not be the major factor involved in strain difference in Gur sensitivity, because another *Tas1r3 *non-taster strain, 129X1/SvJ showed clear Gur sensitivity similar to *Tas1r3 *taster C57BL mice [36], and mice genetically lacking T1r3 still show Gur inhibition of residual responses to sweet compounds, such as glucose and sucrose [25]. Our recent preliminary study using the same cell based assay suggested the possibility that Gur may also interact with the extracellular and transmembrane domains of mouse T1r2 in addition to its major binding site, the extracellular domain of T1r3 (Sanemetsu K, Ninomiya Y., unpublished obervation). The above-mentioned *Tas1r3 *non-taster but Gur-sensitive strain, 129X1/SvJ, possesses the same amino acid composition of the extracellular domain of T1r2 as the C57BL mice, whereas BALB mice possess one amino acid change at position 352 (P352R) [36]. Our previous genotyping by using restriction enzymes indicated that the *Tas1r2 *gene of *dpa CG *may be derived from BALB mice [5]. However, in the previous study, we used only 5 *dpa *CG mice segregated by data from only behavioral taste tests for sweet sensitivity to D-phenylalanine but not by examination of Gur sensitivities in taste nerve responses [5]. So, the data may not be strong enough to rule out the possibility of involvement of the amino acid change of T1r2 (P352R) in mouse strain differences in Gur sensitivity. We are now doing sequence analysis for the *Tas1r2 *gene of *dpa *CG mice. Our previous study demonstrated that mice lacking Gα-gustducin no longer exhibit Gur inhibition [25]. If the T1r2 monomer was involved in the residual sweet responses in mice lacking T1r3, the binding of Gur to the transmembrane domain of T1r2 may affect its interaction with Gα-gustducin, but not with other Gα proteins, and lead to failure of activation of Gα-gustducin by the receptor. With regards to the interaction between receptor and G protein, a recent study in odorant receptor activation and G protein coupling demonstrated that particular mutations of conserved amino acid residues in an intracellular loop or the C-terminus of odorant receptors resulted in loss of activity, without impairing ligand-binding activity, indicating that these residues are involved in coupling to Gαs type G protein [37]. These mutations had, however, little effect on coupling to the promiscuous G protein Gα15, suggesting that coupling of Gαs type G protein and promiscuous G proteins are mechanistically distinct [37]. In the gustatory system, T1r2/T1r3 expressing cells co-express Gα14, Gαi-2, and/or Gαs, in addition Gα-gustducin. Among these G proteins co-expressed with T1r2/T1r3, Gα14 is expressed in the GI circumvallate taste bud cells [20,21]. Therefore, it is of great interest to examine effects of Gur on the interaction between T1r2/T1r3 sweet receptor and these Gα proteins. Collectively, there may be two possible factors involved in strain differences and tongue regional differences in Gur sensitivity. One may relate to the coupling between Gα proteins and sweet receptors, and the other to Gur binding sites of the sweet receptors, especially T1r2, as shown in the speculative model in Fig.5. However, to test these possibilities, future studies are needed.

## Conclusion

The *dpa *CG strain possesses a genetically-increased taste cell population co-expressing T1r2/T1r3 receptors and Gα-gustducin, as compared with its inbred partner BALB strain. Single CT fiber response analysis indicated that the *dpa *CG strain possesses two distinct sweet-responsive fiber populations (GS and GI types) with almost identical sizes, whereas BALB mice have a very small number of GS fibers but a comparable size of GI population, indicating marked increase of GS population in *dpa *CG mice. Thus, the genetically-increased cell population co-expressing T1r2/T1r3/Gα-gustducin in *dpa CG *mice may be associated with an increase of their matched GS type fibers. As shown in the speculative model in Fig.5 Gα-gustducin may be involved in the GS sweet reception pathway and may be a key molecule linking sweet taste receptors and cell type-specific-innervation by their matched fiber class.

## Abbreviations

Gur: gurmarin; CT: chorda tympani; GL: glossopharyngeal; GS:gurmarin-sensitive; GI: gurmarin-insensitive; BALB: BALB/c; C57BL: C57BL/6NCrj; T1r2: taste receptor type1 member 2; T1r3: taste receptor type 1 member 3; D-Phe: D-phenylalanine; *dpa CG*: dpa congenic; β-CD: β-cyclodexrin.

## Authors' contributions

KY, TO, KS and YN recorded single CT nerve fiber responses from *dpa *CG and BALB mice, and analyzed the data. NS, HK and NSako maintained and phenotyped the *dpa *CG strain by using behavioral experiments. YN supervised the electrophysiological and behavioral studies and was instrumental in the conceptual design and analysis of the data. All authors contributed to writing and editing the manuscript.
